# Contrasting In Vitro Flowering Progression Among Three *Phalaenopsis* Hybrids and Developmental Staging in a Miniature Cultivar

**DOI:** 10.3390/plants15213294

**Published:** 2026-10-25

**Authors:** Kuo-Cheng Chang, Ching-Hsueh Wang, Rong-Show Shen

**Affiliations:** 1 Department of Horticultural Science, National Chiayi University, Chiayi City 600355, Taiwan; s1080001@mail.ncyu.edu.tw; 2 Department of Agricultural Technology, National Formosa University, Huwei Township, Yunlin County 632301, Taiwan; chw@nfu.edu.tw

**Keywords:** 6-benzyladenine, anthesis, axillary bud, floral meristem, florogenesis, orchid tissue culture

## Abstract

In vitro flowering permits direct observation of reproductive development, but the progression from visible inflorescence emergence to anthesis remains poorly characterized in *Phalaenopsis*. Here, we compared reproductive development in three commercial hybrids under a standardized culture sequence and developed a preliminary staging scheme for FM1630. Twelve-week-old plantlets were cultured for 12 weeks on modified Vacin and Went medium supplemented with 15 mg L^−1^ 6-benzyladenine (BA), comprising 8 weeks at 20 °C followed by 4 weeks at 25 °C. A BA-free Hyponex No. 1 liquid overlay was then added, and the closed culture vessels were transferred to greenhouse conditions. During the 44-week observation period beginning with the initiation of BA treatment, visible inflorescences emerged in 100% of FM1514, JC2206, and FM1630 plantlets (15/15 for each cultivar). Flower buds formed in 1/15 FM1514, 2/15 JC2206, and 15/15 FM1630 plantlets, whereas anthesis occurred only in FM1630 (2/15; 13.3%). Based on external morphology and 100-µm anatomical sections from independently sampled FM1630 plantlets, we established a preliminary morphological–anatomical staging scheme comprising six major stages (S0–S5). Anatomical observations supported stages S0–S4, whereas S5 was defined by external morphology and subdivided into visible flower-bud expansion (S5a) and anthesis (S5b). The scheme represents a developmental sequence reconstructed from different individuals and provides a provisional reference for sampling comparable developmental stages in FM1630 under the tested culture conditions.

## 1. Introduction

*Phalaenopsis* orchids are economically important ornamental plants whose flowering is strongly influenced by developmental competence and temperature. Commercial plants commonly require cool conditions for spike emergence, whereas axillary buds may remain dormant or develop vegetatively when inductive signals are insufficient [1,2]. At the molecular level, the cooling-responsive floral activator *PaFT1* and its interacting partner *PaFD* promote flowering, whereas the distinct FT-like repressor *PaFTL* participates in age- and temperature-dependent control of spike initiation in *Phalaenopsis aphrodite* [3,4]. These findings place orchid spiking within the broader integration of environmental signals and developmental competence that governs floral induction [5]. In vitro culture provides access to these concealed developmental events and permits repeated observation without destructive removal of whole plants.

**Figure 1:**
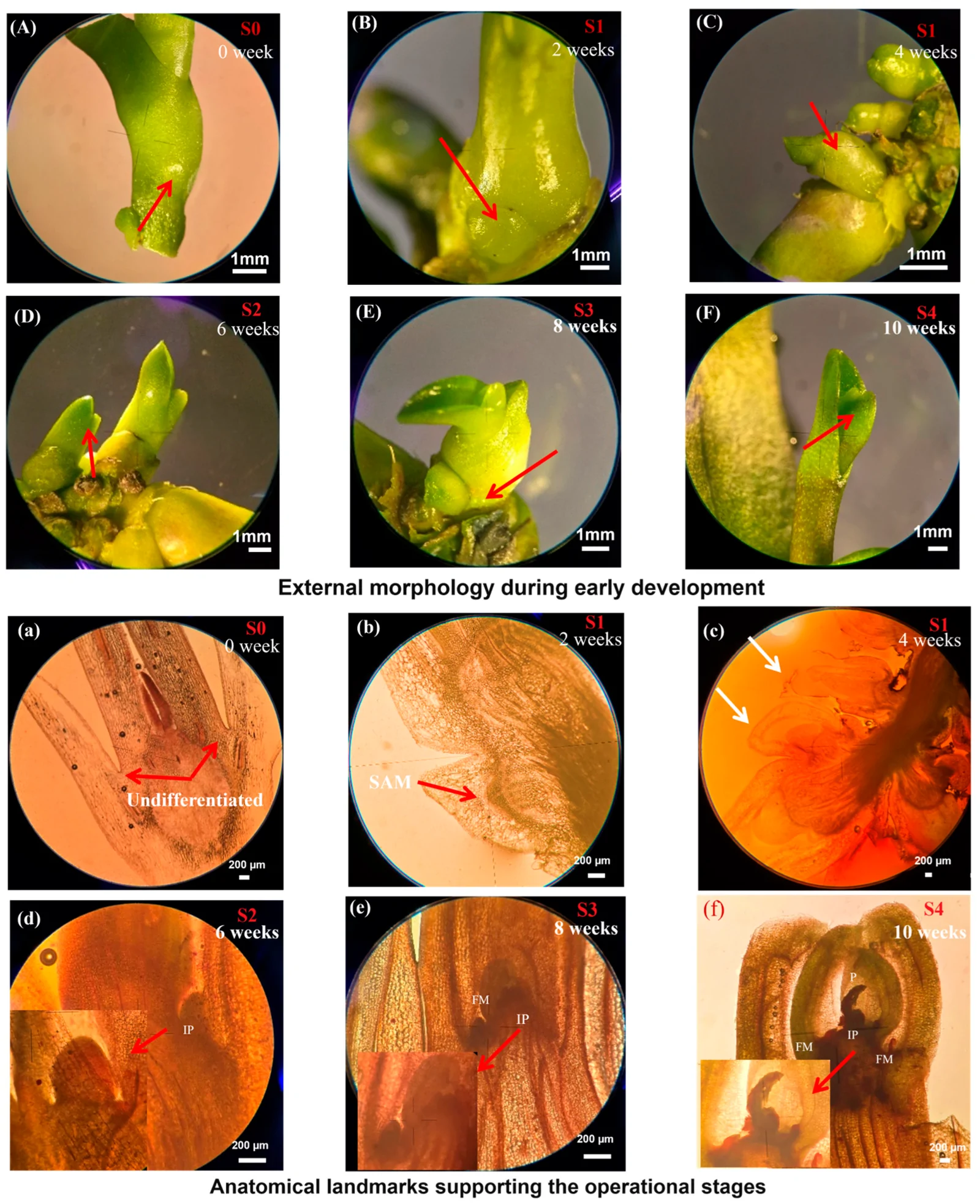

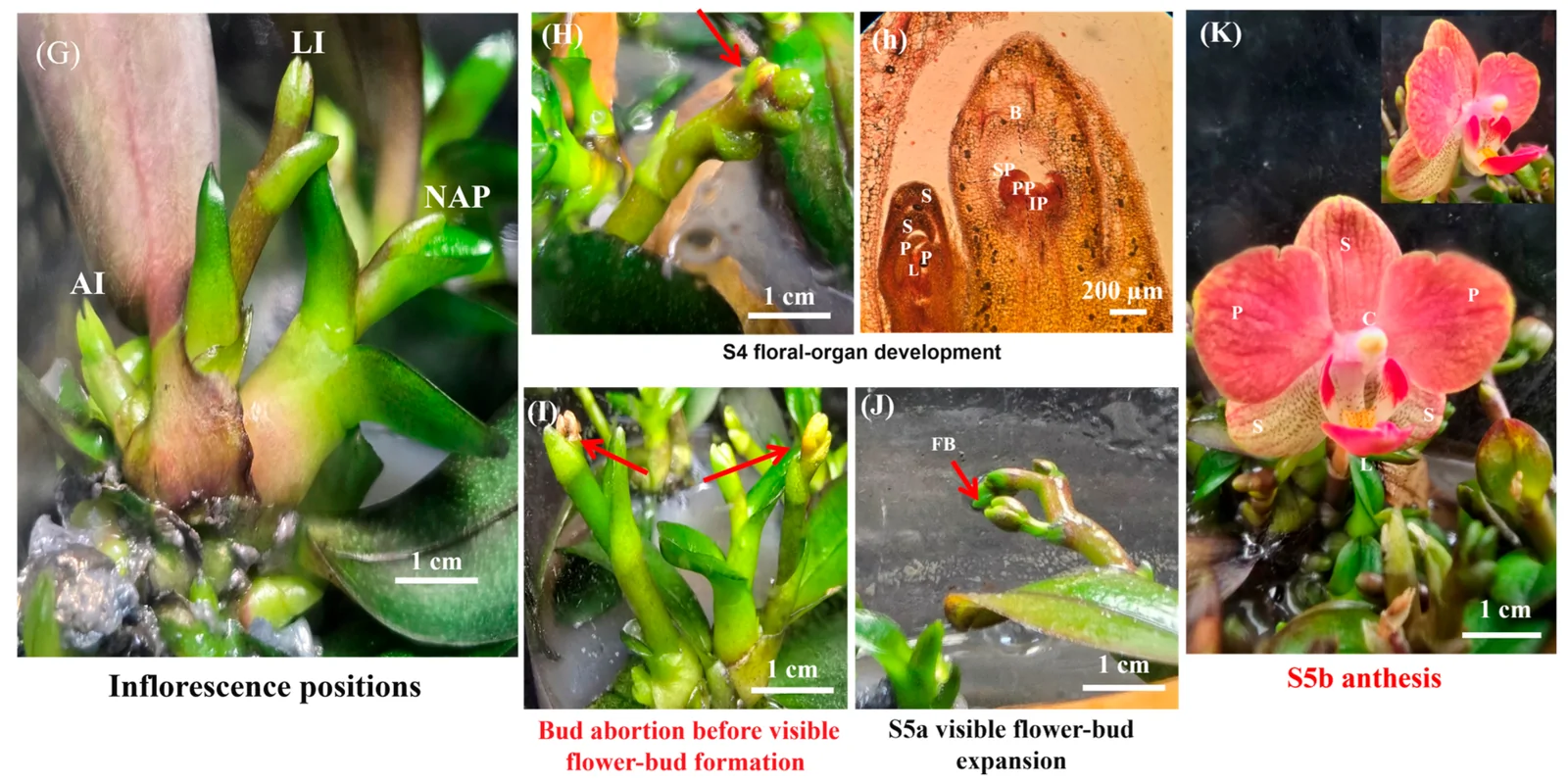
External morphology, anatomical landmarks, and late developmental outcomes supporting a preliminary six-stage operational framework for in vitro florogenesis in *Phalaenopsis* Fangmei Candy Love ‘FM1630’. (**A**–**F**) Representative external views at weeks 0, 2, 4, 6, 8, and 10, assigned to S0, S1, S1, S2, S3, and S4, respectively. These images illustrate observations from different plantlets, not continuous tracking of one individual. Scale bars in (**A**–**F**) = 1 mm. (**a**–**f**) Representative anatomical sections at weeks 0, 2, 4, 6, 8, and 10: a, S0; b and c, S1; d, S2; e, S3; and f, S4. Three separate plantlets from different vessels were sampled at each time point, totaling three plantlets across the three batches rather than three per batch. Weeks 2 and 4 each contributed three plantlets assigned to S1 (six specimens in total). Scale bars in the main anatomical panels (**a**–**f**) = 200 µm; these bars do not apply to the enlarged insets. The insets in (**d**–**f**) are separately acquired higher-magnification photographs of the same specimen and histological section as the corresponding main panel. Calibration records for these photographs were not retained; the insets are provided for qualitative interpretation only. The sequence is a qualitative reconstruction from independent specimens. Additional sections are shown in Supplementary Figure S1. (**G**) Representative inflorescence positions and morphotypes. (**H**,**h**) External and anatomical views at S4. H illustrates small, externally recognizable developing flower buds without conspicuous swelling and provides the flower-bud formation criterion used in Table 2; h illustrates internal floral-organ differentiation. (**I**) Abortion before the developing structure met the external flower-bud formation criterion illustrated in (**H**); this outcome did not qualify as flower-bud formation, did not reach S5a, and was not assigned a separate developmental stage. This external scoring distinction does not imply an absence of prior internal floral differentiation. (**J**) S5a, conspicuous enlargement producing fuller, swollen flower buds that remain unopened. Panels (**H**–**J**) illustrate morphological states or outcomes in different structures and do not establish longitudinal tracking of one individual. (**K**) S5b, successful anthesis, illustrating the terminal flowering substate and differentiated floral organs; the inset shows an oblique flower view. Figure 2 provides plant-context and flower views accompanying the separate descriptive comparison in Table 4. S5a does not necessarily progress to S5b: development may persist, arrest, or undergo apparent abortion. S0, quiescent axillary bud; S1, bud activation and basal enlargement; S2, inflorescence initiation; S3, floral meristem formation; S4, floral organ primordium differentiation. AI, lower axillary inflorescence; NAP, near-apical (uppermost-axillary) inflorescence; B, bract; C, column; FB, flower bud; FM, floral meristem; IP, inflorescence primordium; L, lip; LI, lateral leaf-bearing inflorescence morphotype; P, petal; PP, petal primordium; S, sepal; SAM, shoot apical meristem; SP, sepal primordium. Scale bars in the main external panels (**G**–**K**) = 1 cm; the scale bar in (**h**) = 200 µm. The inset in (**K**) is illustrative and has no scale bar; the main-panel scale bar does not apply to it. In (**h**), L denotes recognizable developing lip tissue, rather than evidence of the earliest lip primordium. In (**K**), L and C indicate the differentiated lip and column at anthesis, not their primordia. Identifiable early lip and column primordia were not captured in the available histological sections; this does not demonstrate their absence during development. Red and white arrows highlight the structures or developmental features described for the corresponding panels; arrow colors provide visual contrast and do not indicate different treatments. Red stage labels indicate the assigned operational stages.

Four related terms are distinguished here. Floral induction denotes acquisition of reproductive competence in response to endogenous and environmental signals. Floral transition is the change in meristem identity from a vegetative or quiescent state to a reproductive state. Florogenesis encompasses development after reproductive commitment through anthesis, whereas floral organogenesis refers specifically to initiation and differentiation of sepals, petals, the lip, and the column. This distinction follows the broader separation of flowering competence, meristem transition, and organ formation in flowering research [5,6,7]. These processes cannot be inferred from external swelling alone; anatomical landmarks are needed to assign reproductive identity. Inflorescence emergence denotes external protrusion of an inflorescence from a leaf axil. Flower-bud formation denotes at least one externally recognizable developing flower bud on a plantlet, using the S4 external morphology illustrated in Figure 1H as the scoring reference. This criterion does not require the conspicuous bud enlargement characteristic of S5a (Figure 1J). Anatomical evidence of floral meristem or floral-organ primordium differentiation alone is insufficient to score this external outcome. Such differentiation does not necessarily lead to externally recognizable flower buds or anthesis; subsequent development may arrest or undergo abortion.

Earlier orchid studies examined the in vitro growth and developmental fates of lateral buds and subsequently demonstrated floral induction and development under in vitro conditions [8,9,10], and more recent transcriptomic studies have resolved hormone-associated spike development, spatial patterning of orchid floral organs, and regulators of axillary-bud dormancy and organ identity [11,12,13,14,15]. Current syntheses emphasize that flowering time, inflorescence initiation, organ identity, floral size, and color in orchids are coordinated by overlapping environmental, hormonal, and transcriptional networks [7,16]. Flower-bud differentiation in mature *Phalaenopsis pulcherrima* has also been divided into six anatomical phases and associated with carbohydrate and hormone dynamics [17], but that study did not follow culture-vessel-grown hybrid plantlets through anthesis. However, a practical staging scheme that connects external morphology with internal meristem and organ landmarks throughout the culture-vessel sequence remains useful for tissue-culture studies. Such a framework can standardize sampling and identify where development arrests after spike initiation.

The present study had three objectives: (1) to compare progression from visible inflorescence emergence to flower-bud formation and anthesis among three commercial *Phalaenopsis* hybrids subjected to a standardized culture sequence; (2) to characterize external morphology and anatomical development in the miniature cultivar *Phalaenopsis* Fangmei Candy Love ‘FM1630’ and construct a preliminary developmental staging scheme; and (3) to provide a descriptive comparison of FM1630 flowers developed in culture vessels with those from mature greenhouse-grown plants. By distinguishing successive reproductive outcomes and relating external morphology to internal developmental landmarks, this study aims to provide a provisional reference for developmental sampling in FM1630 under the tested culture conditions. The staging scheme is reconstructed from independently sampled individuals.

## 2. Results

### 2.1. Contrasting Progression to Floral-Bud Formation and Anthesis

The inflorescence characteristics are summarized in Table 1. Using the prespecified plantlet-level criterion of an inflorescence protruding externally from a leaf axil, 15/15 FM1514, 15/15 JC2206, and 15/15 FM1630 plantlets produced at least one visible inflorescence (Table 2). Exploratory protected LSD comparisons indicated more near-apical inflorescences in JC2206 and FM1630 than in FM1514; the residual-normality departure warrants caution in interpreting these pairwise comparisons. Axillary and total inflorescence numbers differed among all three cultivars and were highest in FM1630, intermediate in JC2206, and lowest in FM1514. Rachis number was higher in FM1630 than in JC2206 and FM1514; the latter two did not differ.

At week 12, immediately before addition of the BA-free Hyponex overlay, regenerated roots were present in every cultivar group. Leaf number was 3.87 ± 0.18, 3.80 ± 1.12, 3.00 ± 0.78 plantlet^−1^ in FM1630, JC2206, and FM1514, respectively (means ± SD of five flask means), with no significant cultivar effect in the one-way ANOVA (*F*(2, 12) = 1.84, *p* = 0.2015). Regenerated-root number was 1.60 ± 0.43, 1.13 ± 1.24, 0.47 ± 0.18 plantlet^−1^, respectively. For regenerated roots, the one-way ANOVA was nonsignificant (*F*(2, 12) = 2.77, *p* = 0.1024), whereas Welch ANOVA (*F*(2, 6.21) = 13.41, *p* = 0.0056) and an exact permutation Kruskal–Wallis test (H = 7.93, *p* = 0.0119) detected cultivar-associated differences. Root-count inference was therefore sensitive to the analysis used; no pairwise significance letters or definitive claim of equal root production are presented. Diagnostic and sensitivity results are provided in Supplementary Table S1.

Later reproductive outcomes differed among the three cultivars (Table 2). Flower-bud formation was scored using the externally recognizable developing-bud morphology illustrated in Figure 1H, without requiring the conspicuous enlargement illustrated in Figure 1J. At least one externally identifiable flower bud formed in 1/15 FM1514 plantlets (6.7%), 2/15 JC2206 plantlets (13.3%), and 15/15 FM1630 plantlets (100%). Neither FM1514 nor JC2206 reached anthesis during the 44-week analysis period. In FM1630, although all 15 plantlets formed visible flower buds, only two produced at least one fully opened flower (2/15; 13.3%). Thus, the high frequency of inflorescence emergence did not translate into a similarly high frequency of visible flower-bud formation in FM1514 or JC2206, or of anthesis in FM1630. These percentages describe plantlet-level outcomes, not the proportion of individual inflorescences or flower buds that completed development.

### 2.2. A Preliminary Six-Stage Framework for In Vitro Florogenesis

External morphology and anatomical sections from independent FM1630 plantlets supported a preliminary six-stage operational framework (S0–S5; Table 3; Figure 1). Because destructive samples were collected from different vessels at 2-week intervals, the stages reconstruct a biologically ordered cross-sectional sequence rather than longitudinal tracking of the same plantlet. The staging material comprised three batches, each with five vessels containing three plantlets per vessel (15 plantlets per batch; 15 vessels and 45 plantlets in total). External development was observed weekly in the remaining, unsampled plantlets. At each time point, a total of three separate plantlets from different vessels were sampled across the three batches (rather than three per batch). Weeks 2 and 4 each contributed three plantlets assigned to S1 (six specimens in total).

External morphology was broadly consistent, and the three anatomical specimens at each sampling time point showed qualitatively similar developmental structures. This qualitative similarity did not establish identical anatomy or validate the stage boundaries. Additional representative sections from the original sampling set are provided in Supplementary Figure S1. Stage assignment relied primarily on external morphology and the available anatomical landmarks. Visually estimated inflorescence lengths and approximate culture times provide supplementary descriptive context only. The length ranges are not validated thresholds and cannot independently determine stage assignment; distributions of measured lengths and stage-onset times were not recorded.

S0 comprised a quiescent axillary bud with no visible elongation. At S1, basal swelling and meristem enlargement indicated bud activation, but external swelling alone was not interpreted as floral induction. At S2, the bud protruded from the leaf axil, and an inflorescence primordium (IP), active cell division, and vascular strands were visible. At S3, a dome-shaped floral meristem (FM) and early floral primordia occurred beneath small bracts. S4 represents floral-organ differentiation, supported by identifiable sepal and petal primordia (Figure 1f,h). Externally, the developing flower buds illustrated in Figure 1H are small and lack the conspicuous swelling seen in Figure 1J. Figure 1H provides the external reference for scoring flower-bud formation in Table 2; anatomical differentiation alone did not meet that scoring criterion. Panel h includes recognizable developing lip tissue (L), but identifiable early lip and column primordia were not captured in the available sections; therefore, their initiation was not established anatomically. The terminal stage S5 was resolved into two externally assessed substates: S5a (conspicuously enlarged, fuller flower buds that remain unopened, as illustrated in Figure 1J; these buds could persist, arrest, or undergo apparent abortion) and S5b (successful anthesis). This subdivision records alternative late outcomes without implying an additional principal developmental stage. Anatomical interpretation was qualitative and based on three specimens per sampling time point for S0–S4, including separate samples at weeks 2 and 4 for S1; S5a and S5b were not sectioned.

**Figure 2:**
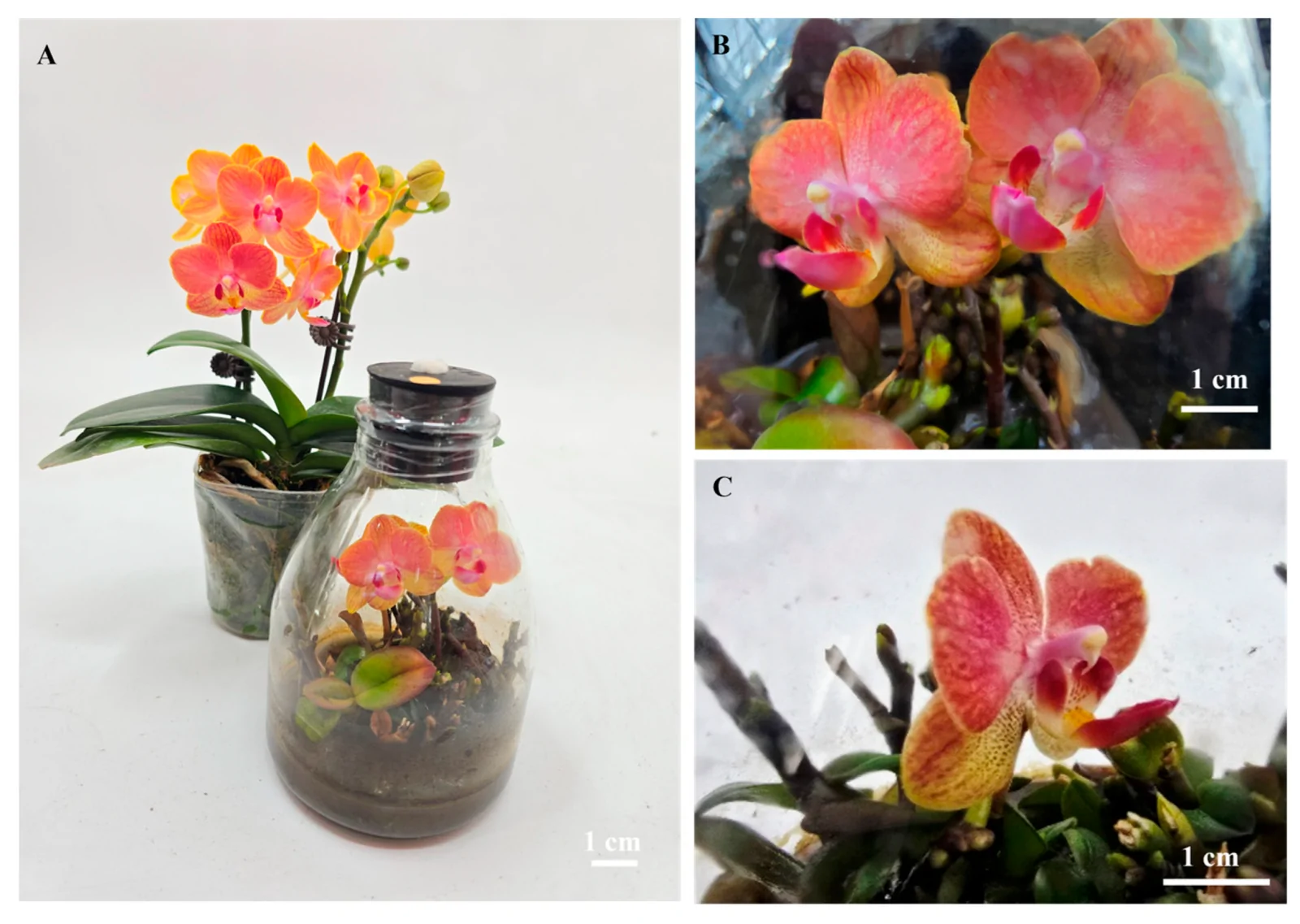
Developmental context of culture-vessel-derived and conventionally greenhouse-grown *Phalaenopsis* Fangmei Candy Love ‘FM1630’ used for the descriptive comparison in Table 4. (**A**) A mature greenhouse-grown plant in a 2.5-inch pot (left/rear) and a flowering plantlet retained inside a culture vessel (right/front). (**B**) Frontal views of culture-vessel-derived flowers. (**C**) An oblique view of a culture-vessel-derived flower. Panel (**A**) illustrates the contrasting plant and culture contexts, and panels (**B**,**C**) provide frontal and oblique views of culture-vessel-derived flowers to accompany the descriptive traits in Table 4. In contrast, Figure 1K documents S5b anthesis and identifies differentiated floral organs. The photographs are illustrative and do not define the sample size reported in Table 4. The groups differed in plant developmental age and preflowering conditions; this figure is not an age-matched experimental comparison. These confounding factors and their implications are discussed in Section 3.5 and Section 3.6. Scale bars in (**A**–**C**) = 1 cm.

Near-apical and axillary inflorescences and total inflorescence number were quantified for all 15 plantlets of each cultivar (Table 1). Lateral inflorescences accompanied by small leaf-like structures were additionally documented as a qualitative morphotype but were not analyzed as a separate quantitative category. These observations indicate morphological variation under the standardized culture sequence but do not establish a treatment effect.

Stage assignment is based primarily on external morphology and the available qualitative anatomical landmarks. Inflorescence lengths are approximate visual estimates provided only as supplementary descriptive references. The listed ranges are not validated classification thresholds and must not be used independently to assign developmental stages. Culture times are likewise descriptive observations under the tested conditions. The staging material comprised three batches, each with five vessels containing three plantlets per vessel (15 plantlets per batch; 15 vessels and 45 plantlets in total). External development was observed weekly in the remaining, unsampled plantlets. The sequence was reconstructed from different plantlets sampled destructively at 2-week intervals. Three separate plantlets from different vessels were selected in total across the three batches at each sampling time point, with one axillary bud or inflorescence apex examined per plantlet and no pooling. S1 included three specimens at week 2 and three at week 4; S5a and S5b were assessed externally and were not sectioned. The flower-bud formation outcome in Table 2 uses the external morphology illustrated in Figure 1H; internal differentiation alone does not qualify. Abortion before this external criterion was met (Figure 1I) was recorded as a failure outcome, not as S5a or an additional stage.

### 2.3. Progression to Anthesis and Developmental Arrest

After the BA-free Hyponex overlay and greenhouse transfer, some inflorescences continued to visible bud formation and expansion, whereas others, including structures that had reached the visible-bud stage, withered or ceased elongation. Figure 1I illustrates abortion before the external flower-bud formation criterion in Figure 1H was met and is distinguished from failure after externally recognizable buds had formed. Because the structures in Figure 1I did not meet the external criterion for S5a, no S5a assignment was made for that panel. Accordingly, S5a represented a morphology-based late developmental state rather than a fixed time interval, and transition from S5a to S5b was not obligatory. The first S5b anthesis occurred at total culture week 36, approximately 23–24 weeks after greenhouse transfer, and flowering was observed from weeks 36 to 40. In FM1630, 2 of 15 plantlets (13.3%) reached S5b. Flowers that reached S5b contained three sepals, two lateral petals, a differentiated lip, a column, and visible pollinia without gross organ loss.

### 2.4. Descriptive Floral Comparison

The anthesis rate of 2/15 plantlets (13.3%) was calculated exclusively from the predefined 12-week-old cultivar-comparison cohort. Floral morphology was evaluated separately using nine intact flowers, one from each of the first nine FM1630 culture vessels to produce an open flower. The sampled material included plantlets initiated at 12, 16, or 20 weeks of age; the number of sampled flowers within each age category was not recorded separately, and no age-specific comparison was performed. One plantlet was selected within each vessel, one inflorescence was selected on each plantlet, and the first flower on that inflorescence was assessed in situ without detachment on the third day after full opening. This separate cohort was not included in the cultivar-level flowering-rate analysis. Culture-vessel-derived and mature greenhouse-grown flowers had the same gross organ complement but differed descriptively in dimensions and RHS color classification (Table 4; Figure 2). Mean flower width was 2.98 cm in the culture-vessel-derived group and 4.12 cm in the greenhouse-grown group, a descriptive difference of 27.8%. The corresponding categorical RHS colors were Orange-Red Group 35B and Red Group 41C.

Dimensional values are means ± sample standard deviation (SD), in cm, from nine flowers per group. Later flowers from the same vessels were not included; subsequent inflorescences were not removed. Dimensional traits and RHS color were assessed on the third day after full flower opening. The culture-vessel-derived group comprised nine intact flowers from nine plantlets in the first nine culture vessels to flower, with one plantlet selected per vessel, one inflorescence selected on each plantlet, and the first flower assessed in situ without detachment. The sampled material included plantlets initiated at 12, 16, or 20 weeks of age; the number of sampled flowers within each age category was not recorded separately, and no age-specific comparison was performed. This separate cohort was not included in the predefined 12-week-old cohort used to calculate flowering rate. The greenhouse group comprised nine flowers from nine mature plants, with one flower assessed per plant. RHS color was visually assigned using the sixth edition (2015) of the RHS Colour Chart under uniform diffuse natural daylight by the same observer at the same adaxial petal position. Culture vessels were not opened for color assessment. No instrumental CIELAB measurement of petals was performed. No inferential comparison is presented because plant age and preflowering environment were not equivalent between groups.

## 3. Discussion

### 3.1. Contrasting Cultivar Responses and the Late-Development Bottleneck

The cultivar comparison distinguishes visible inflorescence emergence from completion of florogenesis. All 15 FM1514 and JC2206 plantlets produced inflorescences, and a small number of plantlets formed flower buds, but neither cultivar reached anthesis. In FM1630, all plantlets formed flower buds and 13.3% reached anthesis. These observations reveal cultivar-associated differences in progression beyond inflorescence emergence under the standardized culture sequence. Recent reviews likewise emphasize that commercial *Phalaenopsis cultivars* differ widely in inflorescence architecture, flower size, and flowering behavior, while the genetic basis of much cultivar-level variation remains unresolved [16].

Among the three cultivars tested, FM1630 showed the most advanced reproductive progression, supporting its selection for further studies of in vitro flowering and developmental staging. Nevertheless, the low anthesis frequency indicates that substantial developmental limitations remain after flower-bud formation and that further optimization is needed. Because only one cultivar represented each flower-size category, the observed response cannot be generalized to miniature or small-flowered *Phalaenopsis* cultivars as a group.

### 3.2. Scope and Value of the Preliminary S0–S5 Framework

This study proposes a preliminary, cultivar-specific framework linking external morphology and anatomical landmarks from a quiescent axillary bud to anthesis in FM1630. Visually estimated inflorescence lengths provide supplementary descriptive context. Earlier work examined the in vitro developmental fates of *Phalaenopsis* lateral buds and demonstrated reproductive development under suitable culture conditions [8,9], while Li et al. [11] used morphological and cytological states to follow early spike development. The present S0–S5 framework extends those observations through floral meristem formation, organ primordium differentiation, visible bud expansion, and anthesis. S5a and S5b are terminal substates that distinguish visible flower-bud expansion from successful opening without adding a seventh principal stage. Its immediate purpose is to support stage-matched sampling under the tested culture sequence. The small number of anatomical specimens and reconstruction from different individuals do not support a universal timetable or a validated staging standard.

The stage terminology reflects the evidence available at each point. S1 records bud activation and basal enlargement, not floral induction, because physiological competence was not assayed. Inflorescence primordium formation at S2 and floral meristem formation at S3 provide progressively stronger morphological evidence of reproductive transition. S4 represents floral organogenesis, with identifiable sepal and petal primordia in the anatomical sections and small developing buds in the external reference view (Figure 1H,h). The external distinction from S5a is the absence of the conspicuous swelling and fuller bud outline illustrated in Figure 1J. Thus, externally recognizable bud formation and subsequent bud enlargement are separate observations. Table 2 uses the Figure 1H criterion for flower-bud formation, whereas S5a requires conspicuous enlargement. S5a accommodates subsequent arrest or apparent abortion, whereas S5b records successful anthesis. Abortion before the Figure 1H external criterion was met (Figure 1I) does not qualify as flower-bud formation or as failure within S5a; this distinction does not exclude prior internal differentiation. Dong et al. [17] described six anatomical phases in mature *P. pulcherrima*, from initial differentiation to column and pollinia differentiation, using 4-µm paraffin sections. The present scheme combines early anatomical observations with externally assessed bud expansion and anthesis in in vitro-grown FM1630 plantlets. The two schemes cover different developmental intervals and should not be matched stage for stage; the temperature responses reported for *P. pulcherrima* also cannot be assumed to apply to FM1630.

### 3.3. Developmental Interpretation and Recent Molecular Evidence

The FM1630 staging observations distinguish early anatomical differentiation from later externally visible reproductive outcomes. Floral meristems and identifiable sepal and petal primordia were observed before the externally assessed terminal substates of visible flower-bud expansion and anthesis. These observations were reconstructed from different individuals and do not establish the subsequent fate of each sectioned structure. Nevertheless, they provide a developmental context for the cultivar-comparison results: all 15 FM1630 plantlets formed visible flower buds, whereas only two reached anthesis. Early reproductive differentiation and visible flower-bud formation therefore should not be treated as evidence that flowering will necessarily be completed.

Previous studies provide context for investigating these developmental steps: flowering regulators and hormone-associated pathways have been examined during spike and axillary-bud development [3,4,15,18,19], and floral-organ identity has been investigated through spatial and functional analyses [12,13,14,20,21]. Lucibelli et al. [22] analyzed separate floral organs and lip substructures from pre-anthesis buds, illustrating the value of organ-specific sampling for future studies. Their sampling stage is not directly equivalent to the stages defined here. In the present study, endogenous hormones, gene expression, and physiological resource status were not measured, and the culture components were not tested independently. The causes of developmental arrest therefore remain unresolved. The preliminary staging scheme may help future studies collect developmentally comparable samples to investigate why some plantlets progress to anthesis while others do not.

### 3.4. Culture Sequence, Liquid Overlay, and Developmental Arrest

For the cultivar comparison, all 12-week-old plantlets received the same culture protocol: culture on modified VW medium containing 15 mg L^−1^ BA for 8 weeks at 20 °C followed by 4 weeks at 25 °C, addition of a BA-free Hyponex No. 1 liquid overlay at culture week 12, and subsequent transfer of the closed culture vessels to greenhouse conditions. Because these culture components were applied sequentially without separate treatment comparisons, their individual contributions to reproductive development cannot be determined from this experiment. Prior studies show that exogenous 6-BA can alter hormone profiles and gene expression during *Phalaenopsis* flower-bud or pedicel development [23,24], but their concentrations, developmental contexts, and sampled tissues differ from those used here. The present data show that inflorescence emergence occurred in all three cultivars under the combined sequence, whereas only 2 of 15 FM1630 plantlets completed anthesis. Factorial experiments are required to identify which culture components influence flowering efficiency.

The Hyponex No. 1 liquid overlay contained sucrose and mineral nutrients and was added at culture week 12 before greenhouse transfer. Because an otherwise comparable treatment without the overlay was not included, its contribution to subsequent flower development cannot be determined. In the present cultivar comparison, all 15 FM1630 plantlets formed externally visible flower buds, but only two reached anthesis. These observations show that visible flower-bud formation did not ensure completion of flowering under the tested conditions; they do not identify the cause of developmental arrest.

New roots were recorded in all three cultivar groups at week 12, before the overlay was added. This observation documents root regeneration following initial root excision, but root function, tissue carbohydrate status, and associations between root regeneration and subsequent bud fate were not measured. Hou et al. [25] examined carbohydrate status and post-shipping performance under simulated dark shipping, a different experimental context from the present in vitro culture system. That study provides context for investigating resource status but does not establish the cause of bud abortion here. Whether root recovery and resource availability influence progression to anthesis remains a hypothesis for future testing. The contribution of the liquid overlay likewise requires a controlled comparison.

### 3.5. Flower Size and Color: Interpretation of the Greenhouse Comparison

Culture-vessel-derived flowers had smaller dimensions and a different categorical RHS color assignment from flowers of mature greenhouse-grown plants. These differences should not be attributed solely to the culture-vessel environment because the descriptive culture-vessel cohort included plantlets starting at 12, 16, or 20 weeks of age, whereas the greenhouse plants had grown for 12 months after deflasking before cool-room forcing. Guan et al. [26] documented substantial petal cell expansion during flower opening in greenhouse-grown *Phalaenopsis* Sogo Yukidian ‘V3’. Their five stages describe unopened buds through fully opened flowers and are not equivalent to the S0–S5 scheme used here. This work supports distinguishing visible bud development from flower opening, but it does not establish the cause of the flower-size differences or developmental arrest observed in FM1630. Recent synthesis also highlights flower size as a complex cultivar trait controlled by interacting developmental and genetic programs [16]. Unequal source capacity, plantlet age, and preflowering history could therefore influence final dimensions without implying abnormal organogenesis.

The contrast between the two cohorts also raises a developmental-acclimation hypothesis relevant to the S5a-to-S5b transition. The mature plants in 2.5-inch pots had established vegetative and root systems and had undergone greenhouse acclimation before flowering, whereas the culture-vessel plantlets were developmentally younger and remained enclosed. Temperature-sensitive blockage of flower development has previously been documented in *Phalaenopsis* [27]. The capacity of the two cohorts to buffer the interaction of irradiance and temperature with the vessel microenvironment, water relations, and the carbohydrate demand of late bud development may therefore have differed, potentially contributing to S5a arrest or apparent abortion. Table 4 is consistent with a difference in developmental context because the flowers that opened inside vessels were smaller, but it does not test this mechanism: only successfully opened flowers were measured, the groups were not age-matched, and environmental exposure and aborted buds were not quantified. This explanation should therefore be treated as a testable hypothesis rather than a demonstrated cause. Controlled comparisons matching genotype and developmental age while varying acclimation, irradiance, temperature, and vessel condition are required.

The RHS Colour Chart provides a standardized horticultural reference for recording plant color, but the assignments in this study are categorical visual observations rather than instrumental measurements of color difference. CIELAB values were not obtained; consequently, the two RHS assignments should not be interpreted as a quantitative color-distance comparison. Transcriptomic evidence links *Phalaenopsis* color variation to secondary-metabolite and flavonoid-pathway genes [28], and tissue-specific flavonoid modules have been identified in the lip [22]. Because pigments and gene expression were not measured here, the RHS classifications are reported only as descriptive phenotypes and are not interpreted mechanistically.

### 3.6. Limitations and Generalizability

The three-cultivar comparison documents contrasting late developmental outcomes among the cultivars tested, but the preliminary S0–S5 framework was developed only in FM1630 and should not be transferred unchanged to other *Phalaenopsis* genotypes. External development was observed weekly, but the anatomical sequence was reconstructed from only three separately selected plantlets per sampling time point at S0–S4 (six S1 specimens across weeks 2 and 4), with no anatomical sections at S5a or S5b. This sampling can illustrate diagnostic landmarks but cannot estimate within-stage variability or validate stable stage boundaries. The separate floral-morphology cohort included plantlets initiated at 12, 16, or 20 weeks of age, whereas greenhouse flowers came from mature plants with a different preflowering history; that comparison is therefore descriptive and cannot isolate an in vitro container effect. The floral sample preferentially represented early-flowering vessels, and the number of flowers in each starting-age category was not recorded separately. Measurements through vessel walls may be affected by optical distortion and flower orientation; no correction for these effects is claimed. The small number of experimental vessels limits assessment of distributional assumptions. Exact rank-based sensitivity tests supported omnibus differences in the four inflorescence/rachis variables and retained a nonsignificant result for leaf number. These omnibus findings do not independently validate the individual LSD pairwise comparisons. Near-apical inflorescence means had zero sample variance in FM1630, and their residual-normality diagnostic indicated a departure. Root-count inference differed among ANOVA, Welch and rank-based analyses. The Table 1 comparisons are exploratory, and the root data do not establish either equal production or a stable pairwise cultivar ranking. Future studies should also assess flower longevity using defined sampling units and standardized observation endpoints. Additional stage-level replication, validation in other genotypes, age-matched flower comparisons, and factorial testing of culture variables are needed before broad generalization or optimization can be claimed.

## 4. Materials and Methods

### 4.1. Plant Material and Experimental Timeline

**Figure 3:**
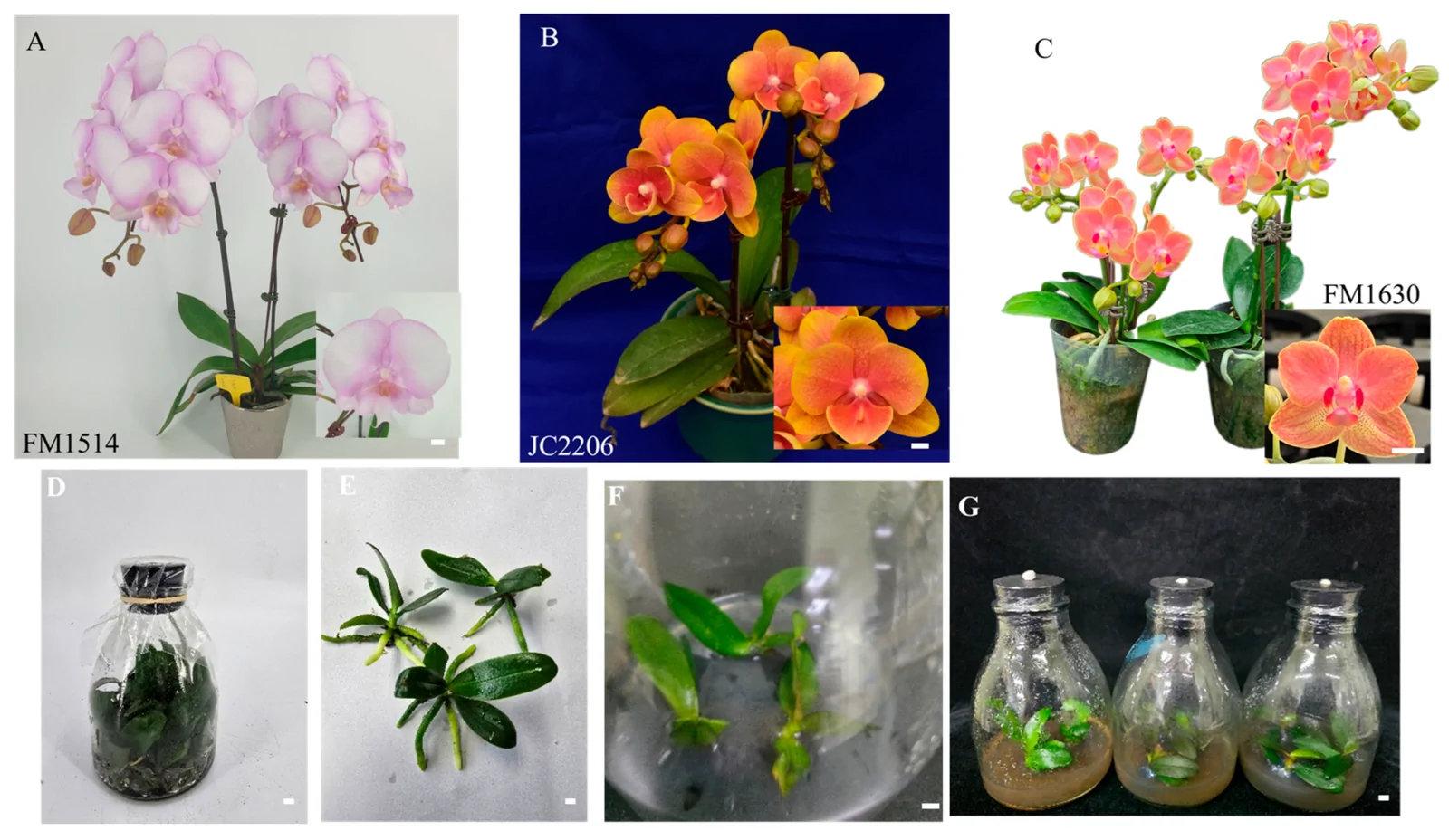
Plant materials and initial experimental procedures. (**A**–**C**) Representative flower-size categories of mature greenhouse-grown plants: (**A**) *Phalaenopsis* Fangmei Dream Angel ‘FM1514’, large-flowered; (**B**) *Phalaenopsis* JC Honey ‘JC2206’, medium-flowered; and (**C**) *Phalaenopsis* Fangmei Candy Love ‘FM1630’, miniature. Insets show representative individual flowers. (**D**) Stock-culture vessel. (**E**) Twelve-week-old plantlets selected for the experiment. (**F**) Plantlets maintained on Hyponex No. 1-based stock-culture medium before selection. (**G**) Selected plantlets transferred to modified VW medium containing 15 mg L^−1^ BA. Scale bars in the insets of (**A**–**C**) and in (**D**–**G**) = 10 mm.

The experiment was initiated on 28 March 2025. The cultivar comparison presented in this manuscript covers the first 44 weeks of culture, during which the first anthesis was observed at week 36 and flowering occurred during weeks 36–40. Monitoring continued until 13 May 2026 to document subsequent flowering events; these later observations were outside the scope of the present analysis and are not reported here. Initial aseptic culture work was conducted at the Floriculture Laboratory (Room A04B-405) and Greenhouse A of the Horticultural Farm, Department of Horticultural Science, National Chiayi University. The subsequent greenhouse phase was conducted at Fangmei Orchid Nursery, Tainan, Taiwan. Healthy, contamination-free in vitro plantlets of three *Phalaenopsis* hybrids were used: the large-flowered *Phalaenopsis* Fangmei Dream Angel ‘FM1514’, the medium-flowered *Phalaenopsis* JC Honey ‘JC2206’, and the miniature *Phalaenopsis* Fangmei Candy Love ‘FM1630’ (Figure 3). For each cultivar, all experimental plantlets were clonally propagated mericlones belonging to the same cultivar line and source batch. The multiplication mother cultures had undergone seven serial subculture passages; these were tissue-culture passages rather than sexual generations. The three cultivars were distinct commercial hybrid genotypes with no known parental relationship and were selected solely to represent contrasting mature flower-size categories (large, medium, and miniature), rather than a shared pedigree. Detailed parental pedigree information was unavailable from the suppliers. This design minimized within-cultivar genetic heterogeneity while enabling a descriptive comparison among the three cultivar lines. Plantlet age was defined as 12 weeks after transfer from intermediate mother cultures to the final stock-culture medium. Plantlets of FM1514 and FM1630 were supplied by Fangmei Orchid Nursery, whereas JC2206 plantlets were supplied by Jincheng Orchid Nursery; both nurseries are located in Tainan, Taiwan. At the beginning of the experiment, uniform plantlets with four fully expanded leaves and at least two well-developed roots were selected. All existing roots were completely excised at the base before the plantlets were transferred to the BA-containing culture medium described below. Complete root excision was a routine preparatory step in this in vitro flowering protocol and reproduced the root-excision component of the induction regime described by Chiu and Chang [10]. In that earlier study, root excision was applied together with BA and low-temperature treatment to promote the transition from vegetative to reproductive growth; because its independent effect was not isolated, root removal is treated here as one component of the standardized culture sequence rather than as an independently tested flowering treatment. Each cultivar was represented by five independent 650-mL culture flasks, with three plantlets per flask (n = 15 plantlets per cultivar; 45 plantlets in total). The plantlets remained inside closed culture vessels throughout the controlled-environment and greenhouse phases.

### 4.2. Culture Medium

The basal medium was a modified Vacin and Went (VW) formulation [29]. Ammonium sulfate [(NH_4_)_2_SO_4_] and potassium nitrate (KNO_3_) were supplied at 250 and 262.5 mg L^−1^, respectively, corresponding to 50% of their concentrations in standard VW medium. The combined molar concentration of these two nitrogen-source salts was 4.49 mM; calculated on an elemental-N basis, the medium contained 6.38 mM inorganic N. The medium contained 25 g L^−1^ sucrose (Taiwan Sugar Corporation, Tainan, Taiwan), 2.0 g L^−1^ peptone (BD Difco, Sparks, MD, USA), 50 g L^−1^ homogenized banana pulp, 200 mL L^−1^ coconut water (Malee COCO; Malee Group Public Company Limited, Pathum Thani, Thailand), 3.0 g L^−1^ Gelrite (PhytoTechnology Laboratories, Lenexa, KS, USA), and 15 mg L^−1^ 6-benzyladenine (BA; Sigma-Aldrich, St. Louis, MO, USA). BA was dissolved in 1 N HCl and autoclaved with the medium. The pH was adjusted to 5.60 ± 0.02 with 1 N NaOH or 1 N HCl. Aliquots of 100 mL were dispensed into 650-mL flasks, capped, and autoclaved at 121 °C for 20 min. The BA concentration was selected on the basis of a preliminary dose-response experiment that was not included in the present cultivar comparison; no claim of optimality is made here.

### 4.3. Culture Sequence and Liquid Overlay

Cultures were maintained at 20 °C for 8 weeks and then at 25 °C for 4 weeks. During the chamber phase, photosynthetic photon flux density was 50 µmol m^−2^ s^−1^ from cool-white fluorescent lamps (FH28D-EX/T, T5-6500 K, 28 W; China Electric Co., Taipei, Taiwan) under a 16-h photoperiod.

At culture week 12, immediately before addition of the liquid overlay, the numbers of leaves and newly regenerated roots were recorded for every plantlet. Regenerated-root number was the count of new roots per plantlet following initial root excision; it was not the percentage of plantlets that rooted. In the original records, the regenerated-root entry was left blank when a plantlet had been examined at week 12 and no regenerated roots were observed. These blanks therefore represented observed counts of zero, not unrecorded or missing observations, and were coded as 0 for analysis. The three plantlet values were averaged within each flask, giving five independent flask means per cultivar. Thereafter, 50 mL of sterile BA-free liquid medium containing 3.0 g L^−1^ Hyponex No. 1 fertilizer (N-P-K 7-6-19; Hyponex Japan Corp., Osaka, Japan) and 25 g L^−1^ sucrose, without Gelrite, was added aseptically to each flask. The liquid medium had been autoclaved at 121 °C for 20 min and cooled before addition. The overlay was included as one component of the standardized culture sequence; its separate effect was not tested.

Immediately after the 12-week chamber phase, vessels were moved to a polyethylene-covered greenhouse at Fangmei Orchid Nursery (23°20′31.49″ N, 120°22′50.89″ E; elevation 17 m). An automatic shade screen was activated when irradiance exceeded 650 µmol m^−2^ s^−1^ and reduced incident light by approximately 60%. Plantlets received natural light, and mean day/night temperatures were maintained at approximately 25/18 °C until anthesis. The plantlets remained inside their culture vessels throughout flower-bud development and anthesis.

### 4.4. Cultivar Comparison and Developmental Outcomes

Cultivar responses were assessed throughout the culture protocol described in Section 4.2 and Section 4.3. Each cultivar comprised five independent culture flasks containing three plantlets each, giving 15 plantlets per cultivar. Culture week 0 was defined as transfer to modified VW medium containing 15 mg L^−1^ BA. Visible inflorescence emergence and inflorescence number were monitored weekly from this transfer onward. All plantlets were included when calculating the proportions showing inflorescence emergence, visible flower-bud formation, and anthesis. Visible inflorescence emergence was recorded when an inflorescence protruded externally from a leaf axil. A near-apical inflorescence was defined as one emerging from the uppermost visible leaf axil immediately below the shoot apex; inflorescences arising from lower leaf axils were recorded as axillary. Near-apical, axillary, and total inflorescence counts in Table 1 represent cumulative formation through week 12, including aborted inflorescences. Rachis number was assessed at week 16. Plantlet-level emergence, visible flower-bud formation and anthesis in Table 2 were recorded through week 44. A rachis was defined as the distal flower-bearing axis of an emerged inflorescence. Flower-bud formation required at least one externally recognizable developing flower bud corresponding to the S4 external morphology illustrated in Figure 1H; conspicuous enlargement at S5a (Figure 1J) was not required. Once a plantlet met this external criterion, it remained included in the cumulative flower-bud formation count, irrespective of subsequent expansion, arrest, abortion, or anthesis. Anatomical differentiation alone and structures that aborted before meeting the Figure 1H criterion (Figure 1I) did not qualify. Anthesis required at least one fully opened flower. The Figure 1H morphology served as the external scoring reference across cultivars; this does not validate the complete FM1630 staging framework in the other cultivars. Each outcome was scored at the plantlet level for FM1514, JC2206, and FM1630.

### 4.5. Developmental Staging and Anatomical Observation

Detailed staging and anatomical observation were conducted in FM1630 because it was the only tested cultivar that completed anthesis. All staging plantlets were 12 weeks old at culture initiation and were maintained separately from the predefined 15-plantlet cultivar-comparison cohort. Destructive samples were collected from different plantlets and culture vessels at culture weeks 0, 2, 4, 6, 8 and 10 after transfer to modified VW medium containing 15 mg L^−1^ BA; consequently, the sequence was reconstructed cross-sectionally rather than followed longitudinally within the same plantlet. The staging material comprised three batches, each with five vessels containing three plantlets per vessel (15 plantlets per batch; 15 vessels and 45 plantlets in total). External development was observed weekly in the remaining, unsampled plantlets. Six preliminary principal stages (S0–S5) were assigned primarily from external morphology and the available anatomical landmarks (Table 3). S4 represents floral-organ differentiation, supported anatomically by identifiable sepal and petal primordia. Figure 1H illustrates its external developing-bud morphology: small recognizable flower buds without the conspicuous swelling characteristic of S5a. Anatomical evidence alone did not qualify as the externally scored flower-bud formation outcome in Table 2. S5 was subdivided into S5a, conspicuous flower-bud enlargement producing a fuller, swollen outline while the flower remained unopened (Figure 1J), and S5b, anthesis of at least one fully opened flower. S5a buds could persist, arrest, or undergo apparent abortion. These substates distinguish bud enlargement from anthesis without adding a seventh principal stage. Structures that aborted before meeting the Figure 1H external criterion (Figure 1I) did not qualify as flower-bud formation and were not assigned to S5a. The inflorescence-length ranges in Table 3 are approximate visual estimates provided only as supplementary descriptive references. They were not derived from systematic length measurements, are not validated classification thresholds, and must not be used independently to assign stages. Approximate culture times are also descriptive; distributions of measured lengths and stage-onset times were not recorded. Three separate plantlets from different vessels were sampled at each time point, totaling three plantlets across the three batches rather than three per batch. Weeks 2 and 4 each contributed three plantlets assigned to S1 (six specimens in total). Each biological specimen consisted of one axillary bud or inflorescence apex from one plantlet; specimens were not pooled. Fresh, unfixed samples were cut into 100-µm sections with a non-freezing DTK-1000 soft-tissue microslicer (Dosaka EM Co., Kyoto, Japan). Sections were immediately stained with 1% acetocarmine and examined using an Olympus SZX12 stereomicroscope (Olympus Corp., Tokyo, Japan) equipped with WHS20X-H/12.5 eyepieces. Images were captured with a UCMOS03100KPA USB 2.0 C-mount camera (part no. TP603100A), coupled through an Olympus U-TV0.5XC-3 adapter, using Image View 64X software (version 3.7.7817). Scale bars in the main anatomical panels were calibrated with a stage micrometer. The separately acquired higher-magnification inset photographs were from the same specimen and section as the corresponding main panel. Calibration records for these inset photographs were not retained; the insets are therefore qualitative illustrations, and the main-panel scale bars do not apply to them. Sections were then transferred to FAA solution (formaldehyde:acetic acid:ethanol = 1:1:18, *v*/*v*/*v*). Additional sections from other plantlets sampled at weeks 6 and 8 are presented in Supplementary Figure S1. These images belong to the original sampling set and do not add biological replicates or constitute independent validation. The week labels indicate the sampling times of the illustrated specimens, not the first occurrence of the structures. Calibration records were not retained for these additional photographs; they are provided for qualitative interpretation only. S5a and S5b were assessed externally and were not sectioned. Anatomical observations were interpreted qualitatively because only three specimens were examined per sampling time point, with six specimens representing S1 across weeks 2 and 4.

### 4.6. Descriptive Comparison of Culture-Vessel-Derived and Greenhouse-Grown Flowers

All flower-color assessments and floral morphological measurements were conducted on the third day after full flower opening. Floral morphology was evaluated using a separate descriptive sample from the first nine culture vessels to flower, each containing three *Phalaenopsis* Fangmei Candy Love ‘FM1630’ plantlets. The sampled material included plantlets initiated at 12, 16, or 20 weeks of age; the number of sampled flowers within each age category was not recorded separately, and no age-specific comparison was performed. One flowering plantlet was selected within each vessel, one inflorescence was selected on each plantlet, and the first intact flower on that inflorescence was assessed in situ without detachment. Thus, the culture-vessel-derived dataset comprised nine flowers from nine plantlets in nine different culture vessels. Subsequent inflorescences remained on the plantlets, but their flowers were excluded from this comparison. This rule limited repeated sampling within vessels; it did not eliminate or test resource competition. This cohort was separate from the predefined 12-week-old cultivar-comparison experiment and was not used to evaluate plantlet-age effects. The culture-vessel-derived flowers were compared descriptively with nine flowers, one from each of nine mature FM1630 plants established in 2.5-inch pots and grown at Fangmei Orchid Nursery. The greenhouse plants had grown for 12 months after deflasking and were subjected to the nursery’s standard cool-room flowering treatment at approximately 25/18 °C day/night. Culture-vessel-derived flowers were measured in situ through the vessel wall using a vernier caliper, without removing the flowers. All original dimensional measurements were recorded in centimetres. Flower width was the span between the outer edges of the two lateral petals; flower length was the distance from the uppermost tip of the dorsal sepal to the lowermost tip of the lateral sepals. Traits recorded were inflorescence and rachis length; flower width and length; dorsal-sepal width and length; petal width and length; lateral-sepal length; and lip width and length. Flower color was visually assigned using the sixth edition (2015) of the RHS Colour Chart under uniform diffuse natural daylight by the same observer at the same adaxial petal position; culture vessels were not opened for color assessment. No instrumental CIELAB measurement of petals was performed. Because the flower groups differed in plant age and preflowering environment, the comparison was descriptive and was not used to attribute differences specifically to the culture-vessel environment.

### 4.7. Data Presentation and Statistical Analysis

For the cultivar comparison, the culture flask was the experimental unit. Production counts were averaged across all three plantlets within each flask, giving five independent flask means per cultivar. Means and sample standard deviations (SD) were calculated across these five values. Original analyses used CoStat 6.451 (Cohort Software, Monterey, CA, USA). During revision, summaries and analyses were recalculated in Python using the author-confirmed record corrections and integer leaf counts detailed in Supplementary Table S1. Regenerated roots had been assessed for every plantlet at culture week 12, before liquid-overlay addition. Under the original recording convention, a blank regenerated-root entry denoted an examined plantlet with no regenerated roots. These entries were therefore coded as observed zeros, not treated as missing observations or replaced by imputed values. All three plantlets, including those with zero regenerated roots, were retained when calculating each flask mean. One-way ANOVA results are reported for the four inflorescence/rachis variables, leaf number, and regenerated-root number. Fisher’s protected least significant difference (LSD) comparisons at α = 0.05 were used only for the four Table 1 variables, whose omnibus ANOVAs were significant. These within-variable comparisons are exploratory; no adjustment across the correlated response variables was made. Residual distributions were assessed using the Anderson–Darling statistic with an approximate normality *p* value, and variance homogeneity using the median-centered Levene (Brown–Forsythe) test. Failure to reject with five flasks per cultivar was not treated as proof of either assumption. Rank-based sensitivity analysis used the tie-corrected Kruskal–Wallis statistic with an exact upper-tail permutation *p* value obtained by enumerating all 756,756 allocations of the 15 flask observations to three labeled groups of five. Flasks, rather than individual plantlets, were permuted. This test evaluates exchangeability under a common distribution and does not by itself identify pairwise mean differences or isolate location differences when distribution shapes differ. Welch ANOVA was also calculated where every group had positive sample variance; it was not calculated for near-apical inflorescences because the FM1630 flask means were all 1.00. All methods and sensitivity results are reported together rather than selecting an analysis based on statistical significance (Supplementary Table S1). Residual-normality diagnostics indicated departures for near-apical inflorescence counts (approximate *p* = 0.0220) and regenerated-root counts (approximate *p* = 0.0012). For near-apical inflorescences, the exact permutation Kruskal–Wallis test also detected an omnibus cultivar difference (*p* = 0.001332), but did not validate the individual LSD comparisons. For regenerated roots, the ANOVA and sensitivity analyses gave different conclusions. Root production is therefore interpreted cautiously, without pairwise significance letters. Emergence time was calculated across all three plantlets within each flask and then summarized across the five equally weighted flask means per cultivar. All 15 plantlets in each cultivar emerged; the corrected record for JC2206 flask 5 plant 3 indicated first visible inflorescence emergence at week 8. Emergence time, plantlet-level emergence, visible flower-bud formation and anthesis (n/N [%]), developmental staging, and anatomical observations were descriptive. Floral measurements in the separate morphology cohort are reported as means ± SD without inferential comparison because age and preflowering environment were unmatched.

## 5. Conclusions

All three *Phalaenopsis* cultivars produced visible inflorescences under the standardized culture sequence, but subsequent development diverged: flower buds formed in 1 of 15 FM1514, 2 of 15 JC2206, and all 15 FM1630 plantlets, whereas anthesis occurred only in FM1630 (2 of 15 plantlets). In FM1630, a preliminary six-stage framework linked external morphology with anatomical evidence for inflorescence initiation, floral meristem formation, and floral-organ differentiation at S0–S4. The terminal S5 was divided into S5a, externally visible flower-bud expansion with possible arrest or apparent abortion, and S5b, successful anthesis. Transition from S5a to S5b was not obligatory under the tested conditions.

These findings apply to the three cultivars and culture conditions examined. The preliminary staging scheme was developed only for FM1630 and reconstructed from different individuals, with three anatomical specimens per sampling time point across S0–S4, with S1 represented at both weeks 2 and 4 and external assessment of S5a and S5b. Additional replication and validation are required before the scheme can be generalized to other genotypes or culture conditions. Because the culture components were not tested independently, their individual effects and optimal levels cannot be determined. The greenhouse-flower comparison remains descriptive because plant developmental age and prior growing conditions were not matched. The small number of independent flasks and the distributional limitations of the count data, exploratory pairwise comparisons, and method-dependent root-count results also limit the statistical conclusions.

## Figures and Tables

**Table 1:** Inflorescence emergence, production, and rachis number in 12-week-old in vitro-cultured *Phalaenopsis hybrid* plantlets under the standardized culture sequence.

Cultivar	Time to Visible Inflorescence Emergence (Weeks)	Near-Apical Inflorescences (no. Plantlet^−1^)	Axillary Inflorescences (no. Plantlet^−1^)	Total Inflorescences (no. Plantlet^−1^)	Rachises (no. Plantlet^−1^)
FM1514	6.60 ± 0.43	0.20 ± 0.30 ^b^	2.20 ± 0.51 ^c^	2.40 ± 0.43 ^c^	1.87 ± 0.80 ^b^
JC2206	6.00 ± 0.91	0.87 ± 0.18 ^a^	4.40 ± 1.48 ^b^	5.27 ± 1.36 ^b^	2.80 ± 1.07 ^b^
FM1630	7.67 ± 0.82	1.00 ± 0.00 ^a^	6.07 ± 1.19 ^a^	7.07 ± 1.19 ^a^	4.47 ± 1.07 ^a^

Near-apical, axillary, and total inflorescence counts are cumulative through week 12 and include aborted inflorescences. Rachis number was assessed at week 16. Values are means ± standard deviation (SD) calculated from five flask means per cultivar (five independent experimental units; three plantlets initially established per flask; 15 plantlets total). Emergence time was calculated based on the three emerged plantlets per flask, and the five flask means were then summarized with equal flask weighting. Emergence time is presented descriptively without significance letters or an inferential test. For count variables, all plantlets were included in the calculations, and different letters within a column indicate differences according to Fisher’s protected least significant difference (LSD) test at *p* < 0.05 following a significant omnibus ANOVA. One-way ANOVA results recalculated from the corrected flask-level count data were: near-apical inflorescences, *F*(2, 12) = 22.55, *p* = 0.000086; axillary inflorescences, *F*(2, 12) = 14.63, *p* = 0.000605; total inflorescences, *F*(2, 12) = 24.05, *p* = 0.000063; and rachises, *F*(2, 12) = 8.87, *p* = 0.0043. These within-variable letter assignments are exploratory and are not adjusted across response variables. Diagnostic and sensitivity analyses are provided in Supplementary Table S1. Near-apical inflorescences arise from the uppermost visible leaf axil; the separate axillary category comprises those arising from lower leaf axils. A rachis is the distal flower-bearing axis of an emerged inflorescence; this variable is distinct from the plantlet-level occurrence of externally visible flower buds reported in Table 2.

**Table 2:** Stage-transition outcomes of three *Phalaenopsis cultivars* under the standardized in vitro flowering sequence.

Cultivar	Inflorescence Emergence, n/N (%)	Flower-Bud Formation, n/N (%)	Anthesis, n/N (%)
FM1514	15/15 (100%)	1/15 (6.7%)	0/15 (0%)
JC2206	15/15 (100%)	2/15 (13.3%)	0/15 (0%)
FM1630	15/15 (100%)	15/15 (100%)	2/15 (13.3%)

Outcomes were recorded through culture week 44. Flower-bud formation required at least one externally recognizable developing flower bud corresponding to the S4 external morphology illustrated in Figure 1H. Conspicuous bud enlargement at S5a (Figure 1J) was not required. Plantlets that subsequently progressed to S5a or anthesis remained included in the flower-bud count. Anatomical differentiation alone and structures that aborted before meeting the Figure 1H criterion (as illustrated in Figure 1I) did not qualify as flower-bud formation. Values represent plantlet counts divided by the total number of plantlets, with percentages in parentheses. Each cultivar group comprised five independent flasks with three plantlets per flask (15 plantlets total per cultivar). Outcomes are presented descriptively without inferential testing.

**Table 3:** Preliminary six-stage operational framework for in vitro florogenesis in *Phalaenopsis* Fangmei Candy Love ‘FM1630’, with terminal substates S5a and S5b. Culture times are observations under the standardized sequence used in this study.

Stage	Approximate Visually Estimated Length	Approximate Time and Condition	External Criterion	Anatomical Landmark
S0	0 mm	Week 0	Quiescent axillary bud concealed at leaf axil	Undifferentiated axillary meristem
S1	1–5 mm	Weeks 2–4; 20 °C	Basal swelling and early bud activation	Meristem enlargement and early vascular differentiation
S2	6–15 mm	Weeks 4–6; 20 °C	Bud protrusion and early inflorescence elongation	Inflorescence primordium, active cell division, and vascular strands
S3	16–25 mm	Weeks 6–8; 20 °C	Elongating apex subtended by small bracts	Dome-shaped floral meristem and early flower primordia
S4	26–35 mm	Weeks 9–12; 25 °C	Small, externally recognizable developing flower buds without conspicuous swelling (Figure 1H)	Sepal and petal primordia; lip and column primordia not captured in the available sections
S5a	>35 mm	After week 12; greenhouse phase; variable duration	Conspicuously enlarged, fuller flower buds that remain unopened (Figure 1J); possible persistence, arrest, or apparent abortion	Not assessed anatomically
S5b	—	Weeks 36–40 total culture; greenhouse phase	Anthesis of at least one fully opened flower	Not sectioned; complete floral organs and visible pollinia confirmed externally

**Table 4:** Descriptive floral traits of a separate mixed-starting-age cohort of culture-vessel-derived and mature greenhouse-grown *Phalaenopsis* Fangmei Candy Love ‘FM1630’ flowers.

Trait	Culture-Vessel-Derived Flowers (n = 9)	Mature Greenhouse-Grown Flowers (n = 9)
Inflorescence length (cm)	4.89 ± 0.76	19.61 ± 4.08
Rachis length (cm)	1.83 ± 0.89	6.78 ± 0.67
RHS color	Orange-Red Group 35B	Red Group 41C
Flower width (cm)	2.98 ± 0.32	4.12 ± 0.10
Flower length (cm)	2.66 ± 0.21	3.84 ± 0.09
Dorsal sepal width (cm)	1.39 ± 0.11	1.93 ± 0.13
Dorsal sepal length (cm)	1.37 ± 0.10	1.76 ± 0.12
Petal width (cm)	1.27 ± 0.10	1.79 ± 0.19
Petal length (cm)	1.37 ± 0.11	1.86 ± 0.07
Lateral sepal length (cm)	1.47 ± 0.09	1.70 ± 0.10
Lip width (cm)	0.79 ± 0.09	0.91 ± 0.08
Lip length (cm)	1.10 ± 0.23	1.81 ± 0.06

## Data Availability

The flask-level count data and statistical results used for the cultivar comparison are provided in Supplementary Table S1. Additional data and analysis records are available from the corresponding author upon reasonable request.

## Supplementary Materials

The following supporting information can be downloaded at: https://www.mdpi.com/article/10.3390/plants15213294/s1. Table S1: Statistical analyses of the three cultivar comparison; Figure S1: Additional representative sections from FM1630 plantlets sampled at culture weeks 6 and 8.
