# Supplementary material for: Contrasting In Vitro Flowering Progression Among Three Phalaenopsis Hybrids and Developmental Staging in a Miniature Cultivar
Source: Plants (Basel). 2026 Oct 25;15(21):3294. doi: 10.3390/plants15213294 (PMC13765575; doi:10.3390/plants15213294)
Supplement: Supplementary file 1 [file plants-15-03294-s001.zip › plants-4589823-supplementary.pdf]

Supplementary Table S1

Statistical analyses of the three cultivar comparison

Each cultivar contributed five independent culture flasks containing three plantlets. All analyses below use flask means (15 independent observations), with sample SD across flasks. Inflorescence counts were cumulative through week 12, including aborted inflorescences; rachises were counted at week 16. Leaf and regenerated-root counts were recorded for every plantlet at culture week 12, before liquid-overlay addition. In the original records, a blank regenerated-root entry indicated that the plantlet had been examined and no regenerated roots were observed. Such entries were coded as observed zeros, not as missing data or imputed values. Zero counts were retained when averaging the three plantlets within each flask. Corrected values follow the authors' record clarifications during revision.

A Omnibus ANOVA and diagnostic checks

Variable	F (2,12)	ANOVA P	AD A^{2*}	AD approx. P	BF F(2,12)	BF P
Near-apical inflorescences	22.5455	0.000086	0.8976	0.0220	1.2727	0.3153
Axillary inflorescences	14.6340	0.000605	0.2593	0.7137	0.6224	0.5531
Total inflorescences	24.0450	0.000063	0.4500	0.2760	0.9347	0.4195
Rachises	8.8712	0.0043	0.3002	0.5819	0.1077	0.8988
Leaves	1.8363	0.2015	0.5862	0.1268	1.6854	0.2264
Regenerated roots	2.7722	0.1024	1.4159	0.0012	0.5493	0.5912

AD: Anderson–Darling residual-normality diagnostic; A^{2*} is the finite-sample-adjusted statistic and P is approximate. BF: median-centered Levene (Brown–Forsythe) variance test. With five flasks per cultivar, nonsignificant diagnostics do not establish normality or homogeneous variances. All diagnostic P values are reported without multiplicity adjustment.

B Sensitivity analyses

Variable	KW H	Exact KW P	Welch F (df1, df2)	Welch P
Near-apical inflorescences	11.0624	0.0013	Not calculated	—
Axillary inflorescences	9.9942	0.000959	22.8009 (2, 6.58)	0.0011
Total inflorescences	11.3011	0.000095	36.8079 (2, 6.37)	0.000315
Rachises	8.4911	0.0062	8.7064 (2, 7.83)	0.0102
Leaves	5.1196	0.0723	2.6114 (2, 5.74)	0.1562
Regenerated roots	7.9262	0.0119	13.4051 (2, 6.21)	0.0056

KW: tie-corrected Kruskal–Wallis statistic. Exact P is the proportion of all 756,756 labeled 5/5/5 allocations with H at least as large as observed. No Monte Carlo sampling was used. Permutation assumes exchangeability under a common-distribution null; it is not a test of means alone. Welch was not calculated for near-apical inflorescences because FM1630 had zero sample variance.

Interpretation and pairwise comparisons

The four Table 1 variables had significant omnibus ANOVA and exact KW results. Fisher's protected LSD was applied only to these four variables. The letters below were recalculated from the corrected records and remain within-variable exploratory comparisons, with no adjustment across correlated outcomes; exact omnibus KW results do not validate each pairwise LSD contrast. Near-apical counts showed a residual-normality departure (AD approximate P = 0.0220), while their exact KW result remained significant (P = 0.001332). The near-apical LSD letters should therefore be interpreted cautiously.

Variable	LSD at 0.05	FM1514	JC2206	FM1630
Near-apical inflorescences	0.2781	b	a	a
Axillary inflorescences	1.5622	c	b	a
Total inflorescences	1.4789	c	b	a
Rachises	1.3626	b	b	a

Leaf-number inference was nonsignificant in ANOVA (P = 0.2015), Welch ANOVA (P = 0.1562) and exact KW testing (P = 0.0723). No leaf-number pairwise letters are assigned.

Regenerated-root results were method-dependent. ANOVA gave P = 0.1024; Welch gave P = 0.0056 and exact KW P = 0.0119. The residual AD diagnostic gave approximate P = 0.0012, although the variance diagnostic was nonsignificant (P = 0.5912). The first JC2206 flask had 3.33 roots per plantlet, whereas its other four flask means were 0.67, 0.33, 0.67 and 0.67. This observed flask was retained. The different weighting and rank dependence of the methods contribute to their different results. The rank result concerns distributions, and should not be interpreted as proving a particular mean or median difference when distribution shapes differ. Root means and SD are reported descriptively; no root pairwise letters, equality claim or definitive cultivar ranking is assigned.

Poisson or negative-binomial models were not fitted to the fractional flask means as if they were individual integer counts. The revision uses flask-level rank and Welch sensitivity analyses to preserve the experimental unit. All sensitivity analyses were added during revision and were not prespecified confirmatory tests.

Calculation and verification

Revision-stage computations used local Python functions. ANOVA sums of squares and F statistics were independently checked using a NumPy least-squares model. Tied ranks were assigned to equal flask totals; double ranks were represented as integers for exact tail comparisons. The exact procedure enumerates assignments of distinct flask identities, retaining equal-valued observations as separate experimental units. No plantlet was treated as an independent replicate for these tests.

C Flask means used for analysis

Cultivar	Flask	Near-apical	Axillary	Total	Rachises	Leaves	New roots
FM1514	1	0.0000	1.6667	1.6667	1.0000	3.0000	0.6667
FM1514	2	0.0000	2.6667	2.6667	2.6667	3.3333	0.3333
FM1514	3	0.6667	1.6667	2.3333	2.3333	3.6667	0.6667
FM1514	4	0.3333	2.3333	2.6667	1.0000	1.6667	0.3333
FM1514	5	0.0000	2.6667	2.6667	2.3333	3.3333	0.3333
JC2206	1	0.6667	5.6667	6.3333	1.6667	5.3333	3.3333
JC2206	2	0.6667	5.3333	6.0000	2.6667	4.3333	0.6667
JC2206	3	1.0000	5.3333	6.3333	4.3333	3.6667	0.3333
JC2206	4	1.0000	3.3333	4.3333	3.3333	3.3333	0.6667
JC2206	5	1.0000	2.3333	3.3333	2.0000	2.3333	0.6667
FM1630	1	1.0000	6.0000	7.0000	4.3333	3.6667	1.3333
FM1630	2	1.0000	6.0000	7.0000	4.3333	4.0000	1.6667
FM1630	3	1.0000	4.3333	5.3333	3.0000	4.0000	2.3333
FM1630	4	1.0000	6.3333	7.3333	4.6667	3.6667	1.3333
FM1630	5	1.0000	7.6667	8.6667	6.0000	4.0000	1.3333

Units are counts per plantlet, averaged over three plantlets within each flask. Calculations used full precision, not the rounded values shown here. All 15 plantlets per cultivar emerged. Emergence time was averaged across all three plantlets within each flask, then across the five equally weighted flask means. JC2206 flask means were 5.0000, 6.3333, 6.0000, 5.3333 and 7.3333 weeks (cultivar mean ± SD: 6.00 ± 0.91 weeks). Emergence time remains descriptive and was not subjected to inferential testing.

Record corrections used during revision

JC2206 flask 5 plant 1: total inflorescences 4 (= 1 near-apical + 3 axillary). JC2206 flask 5 plant 3: first visible inflorescence at week 8; near-apical 1, axillary 2 and total inflorescences 3 through week 12; rachises 0 at week 16. This author-confirmed clarification supersedes the earlier classification as non-emerging and changes the emergence outcome to 15/15 (100%). FM1630 flask 3 plant 3: near-apical 1 and total 4 (= 1 + 3). FM1514 flask 5 rachises: 4, 2 and 1, yielding 7/3 per plantlet. Blank regenerated-root cells in the original plantlet records denoted observed counts of zero at the week-12 assessment, as confirmed by the author; they did not represent unrecorded or missing observations. Coding these entries as 0 preserved the original observations. Leaf values follow the author-confirmed integer records. These are corrections to the analysis data; they do not add biological replicates.

Supplementary Figure S1

Panel A   Week 6

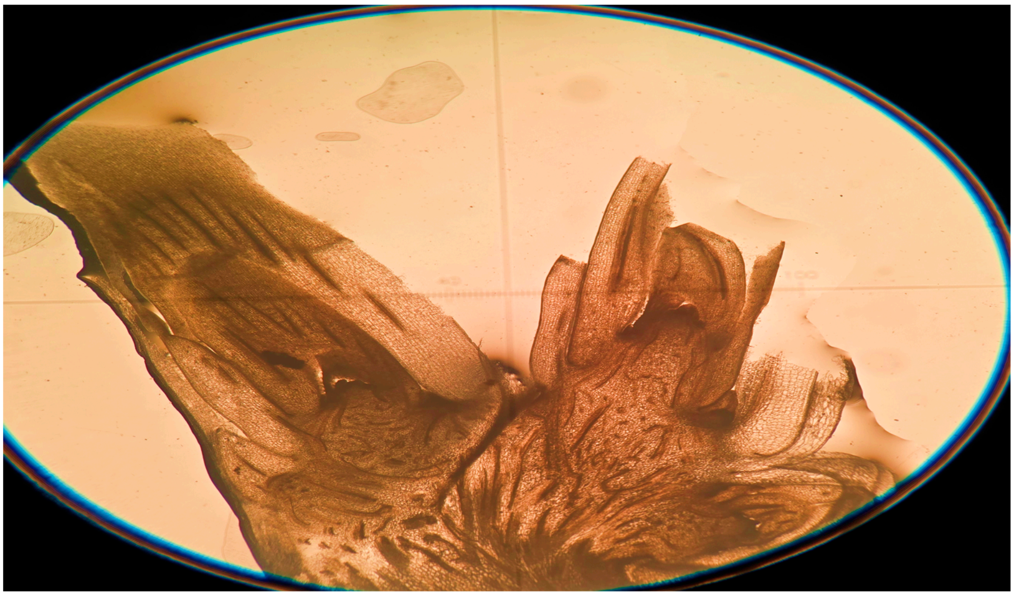

(A) Additional representative section from an FM1630 plantlet sampled at culture week 6. Panels A, B and C represent separate plantlets from the original sampling set, different from those illustrated in Figure 1. Different section planes are shown. Sampling times are not stage-onset times. Calibration records were not retained; the ocular divisions are not calibrated physical units. Images are qualitative, are not shown at a common scale and do not increase biological replication or independently validate the stages.

Supplementary Figure S1 continued

Panel B   Week 8

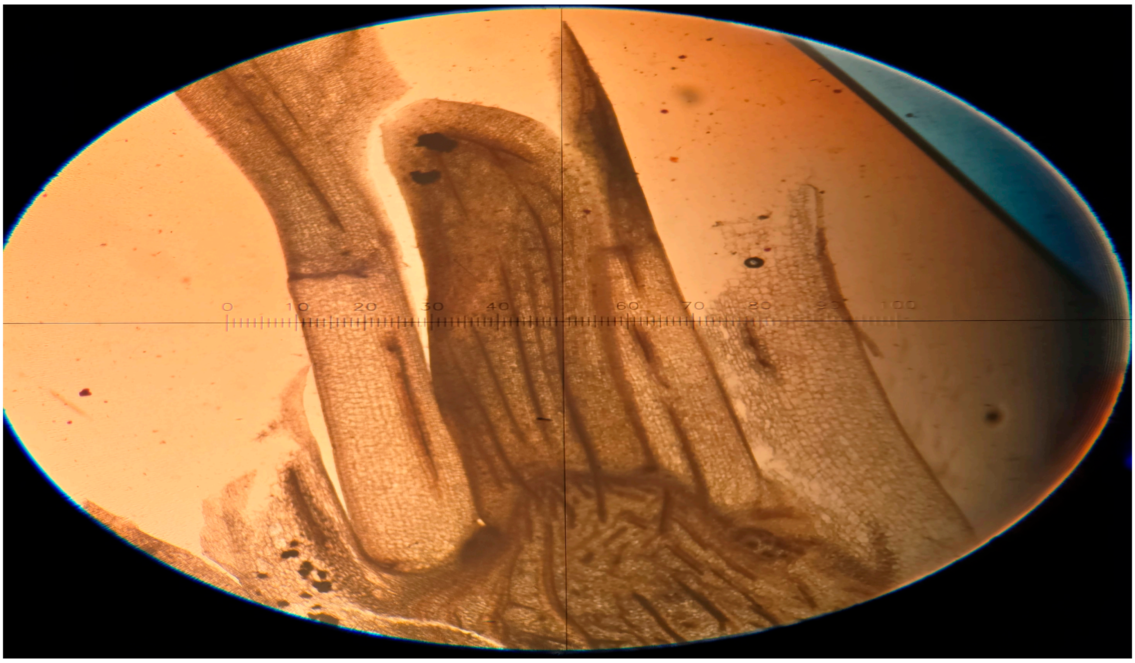

(B) Additional representative section from an FM1630 plantlet sampled at culture week 8. Panels A, B and C represent separate plantlets from the original sampling set, different from those illustrated in Figure 1. Different section planes are shown. Sampling times are not stage-onset times. Calibration records were not retained; the ocular divisions are not calibrated physical units. Images are qualitative, are not shown at a common scale and do not increase biological replication or independently validate the stages.

Supplementary Figure S1 continued

Panel C   Week 8

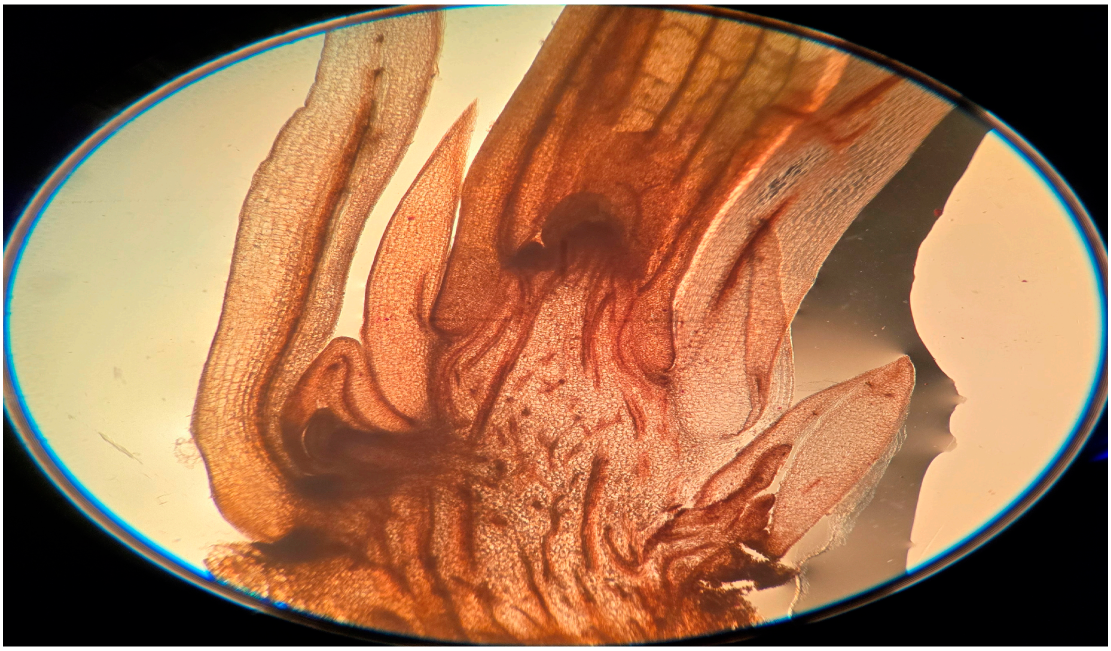

(C) Additional representative section from an FM1630 plantlet sampled at culture week 8. Panels A, B and C represent separate plantlets from the original sampling set, different from those illustrated in Figure 1. Different section planes are shown. Sampling times are not stage-onset times. Calibration records were not retained; the ocular divisions are not calibrated physical units. Images are qualitative, are not shown at a common scale and do not increase biological replication or independently validate the stages.
